# Semi-automatic translation of medicine usage data (in Dutch, free-text) from Lifelines COVID-19 questionnaires to ATC codes

**DOI:** 10.1093/database/baad019

**Published:** 2023-04-26

**Authors:** Alexander J Kellmann, Pauline Lanting, Lude Franke, Esther J van Enckevort, Morris A Swertz

**Affiliations:** Department of Genetics, Genomics Coordination Center UMCG / University of Groningen, Antonius Deusinglaan 1, Groningen 9713 AV, The Netherlands; Department of Genetics, University Medical Center Groningen, Antonius Deusinglaan 1, Groningen 9713 AV, The Netherlands; Department of Genetics, University Medical Center Groningen, Antonius Deusinglaan 1, Groningen 9713 AV, The Netherlands; Oncode Institute Office Jaarbeurs Innovation Mile (JIM), Jaarbeursplein, Utrecht 63521 AL, The Netherlands; Department of Genetics, Genomics Coordination Center UMCG / University of Groningen, Antonius Deusinglaan 1, Groningen 9713 AV, The Netherlands; Department of Genetics, Genomics Coordination Center UMCG / University of Groningen, Antonius Deusinglaan 1, Groningen 9713 AV, The Netherlands

## Abstract

The mapping of human-entered data to codified data formats that can be analysed is a common problem across medical research and health care. To identify risk and protective factors for severe acute respiratory syndrome coronavirus 2 (SARS-CoV-2) susceptibility and coronavirus disease 2019 (COVID-19) severity, frequent questionnaires were sent out to participants of the Lifelines Cohort Study starting 30 March 2020. Because specific drugs were suspected COVID-19 risk factors, the questionnaires contained multiple-choice questions about commonly used drugs and open-ended questions to capture all other drugs used. To classify and evaluate the effects of those drugs and group participants taking similar drugs, the free-text answers needed to be translated into standard Anatomical Therapeutic Chemical (ATC) codes. This translation includes handling misspelt drug names, brand names, comments or multiple drugs listed in one line that would prevent a computer from finding these terms in a simple lookup table. In the past, the translation of free-text responses to ATC codes was time-intensive manual labour for experts. To reduce the amount of manual curation required, we developed a method for the semi-automated recoding of the free-text questionnaire responses into ATC codes suitable for further analysis. For this purpose, we built an ontology containing the Dutch drug names linked to their respective ATC code(s). In addition, we designed a semi-automated process that builds upon the Molgenis method SORTA to map the responses to ATC codes. This method can be applied to support the encoding of free-text responses to facilitate the evaluation, categorization and filtering of free-text responses. Our semi-automatic approach to coding of drugs using SORTA turned out to be more than two times faster than current manual approaches to performing this activity.

**Database URL** https://doi.org/10.1093/database/baad019

## Introduction

The mapping of human-entered data to codified data formats that can be analysed is a common problem across medical research and health care. To make medication data in free-text entries accessible and analytically useful for computational processing, they can be encoded with standard identifiers. For example, to evaluate whether a drug influences the severe acute respiratory syndrome coronavirus type 2 (SARS-CoV-2) susceptibility requires statistical analysis. Standard identifiers can be used to automatically group, categorize, count and filter similar drugs. The Molgenis plugin SORTA can support an expert in mapping free-text terms to terms within an ontology, but there is still a shortage of ontologies for commonly used terms in languages other than English. Faced with this scenario within the context of Lifelines COVID-19 questionnaire programme, we developed a method and an ontology to reduce the amount of manual curation required for a very large dataset.

In response to the COVID-19 pandemic, the Lifelines COVID-19 Cohort was initiated within the Lifelines prospective follow-up biobank, a project following >160 000 people in the Northern Netherlands (https://www.lifelines.nl/) (1). Online questionnaires were sent out to participants starting 30 March 2020, with the aim of identifying risk and protective factors for SARS-CoV-2 susceptibility and COVID-19 severity, as well as socio-psychological impacts of the pandemic (2). Since the use of specific drugs was thought to be a potential risk factor, questionnaires included both multiple-choice and open-ended questions about participant drug use in previous week(s) (Supplementary Material Attachment 5). For analysis, the drugs should then be grouped into suitable categories and filtered accordingly. This could be done using standard taxonomies. The Anatomical Therapeutic Chemical (ATC) Classification System is a widely used coding system to classify drugs, which is recommended by the World Health Organization (WHO) (https://www.who.int/tools/atc-ddd-toolkit/atc-classification). It classifies drugs by their use and is therefore effective when it comes to filtering and statistics.

In previous Lifelines studies, drug usage was registered by a staff member during an on-site visit (3). However, participants in the COVID-19 study filled in this information themselves, and the open questions were added because the online survey tool did not allow for full drug databases to provide answer options to Lifelines participants. While various spelling mistakes and separators were to be expected, an online survey was necessary to keep participants and staff safe during the pandemic. The challenge provided by the free-text drug usage questions, however, was the large number of responses. Thousands of participants received the questionnaires, and a notable proportion of responses concern drugs that were not predefined, which made manual curation of the free-text responses unfeasible. It is a relatively simple task for a computer to use a database to look up the ATC codes for drugs when those are written in exactly the same way. However, when drugs are misspelt, or when additional information is given, assigning ATC codes to free-text answers can be time-consuming manual work that requires pharmacological expertise. To reduce the time repetitively spent on the manual mapping of thousands of free-text answers, we thought about how to support these experts. Our aim is to provide a method to accelerate this process.

Currently, many tools for text recognition exist. For example, named entity recognition tools like the Natural Language Toolkit (https://www.nltk.org) or spaCy (https://spacy.io) can help with tokenization, text classification, tagging parts of speech and removal of stop words. Therefore, they often rely on large corpora of stop words in different languages. Various approaches exist to extract information from free text, from natural language processing to text mining and classification (4). There are also more specific tools for drug name recognition, ranging from simple dictionary-based approaches to more complex approaches using machine learning and ontologies (5, 6). Spell checkers may also be helpful when dealing with misspelt terms (7).

As all of these methods needed adaptation to fit our needs and we did not want to rely on a fully automatic matching tool like LexMapr (8), we preferred to work with familiar, in-house-developed software. We therefore extended SORTA, a previously developed Molgenis plugin for semi-automatic matching of free-text terms to an ontology (9). MOLGENIS is an open source platform for FAIR scientific data (10). SORTA uses a combination of Apache Lucene (https://lucene.apache.org/) and N-gram to match non-standard input terms to ontology terms. This combination allows it to recognize Synonyms specified in the ontology and even recognize terms that contain minor spelling mistakes. For each term, SORTA generates a similarity score and shows a selection of the best-matching results as suggestions, allowing the expert to choose the best fit and thereby drastically reducing the time spent on the task.

## Materials and methods

In the first week of the project, 42 539 Lifelines participants took part in the Lifelines COVID-19 survey. The survey includes 10 questions about drug use [for a list of these questions, see the Supplementary Material Attachments or the Lifelines Wiki (https://wiki-lifelines.web.rug.nl/doku.php?id=medication_covid-19)]. The survey section on drug usage starts with a question about whether the participant has taken any drugs used to treat common health conditions, such as blood pressure–lowering or diabetes-related drugs. The subsequent nine drug-related question sections are about these different common classes of drugs. Each section has a multiple-choice selection of commonly used drugs and a free-text box for participants to enter additional drug names. In the resulting dataset, we were confronted with a list of Dutch drug names in the free-text answer fields. These often described well-known drugs with generic names, but many answers contained problems such as spelling mistakes, brand names and use of different separators. We therefore developed a method to semi-automatically convert the Dutch free-text medication data to standard ATC codes and applied this to a subset of the COVID-19 questionnaire dataset.

### Process overview

**Figure 1.**
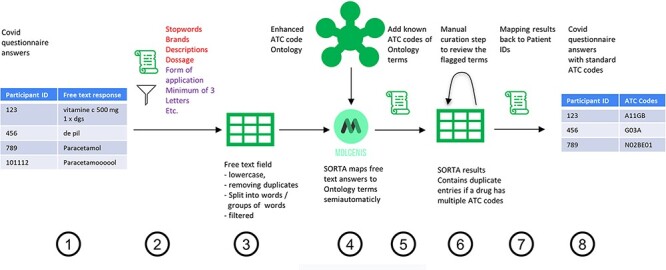
Process overview on how to get from the free-text answers to ATC codes. We pre-processed the COVID-19 questionnaire answers (Step 1) by first removing participant IDs to anonymize the data, then removing duplicates and unnecessary information and splitting the answer into single terms (Step 2). The result is a list of slightly filtered, unique free-text answers (Step 3). Expert users can then use SORTA to assign ontology terms to each free-text answer (Step 4). Based on a confidence threshold, users can automatically accept assignments. The extended SORTA 2.0 user interface also has the option to flag terms for manual curation, to fix less confident assignments afterwards. After downloading SORTA results, a script can automatically assign the ATC codes (Step 5) to the ontology terms. The cases that could not be solved within SORTA and therefore were flagged for manual curation can be processed by a human expert (Step 6). After manual curation of the more complex cases, the final step (Step 7) recombines the ATC codes with the participant IDs to produce a table with participant IDs and corresponding medication’s standardized ATC code(s) (Step 8).

To start, SORTA needs an ontology containing drug names with their respective ATC codes as a knowledge base and the free-text answers as input terms to match free-text inputs to ATC codes. Figure 1 shows the overall process of assigning the free-text answer with ATC codes, which includes eight steps: pre-processing (Steps 1–3), curation within SORTA (Steps 4 and 5), manual curation of difficult cases (Step 6) and recombining ATC codes with participant IDs (Steps 7 and 8).

### Anonymizing answers to respect privacy

**Figure 2.**
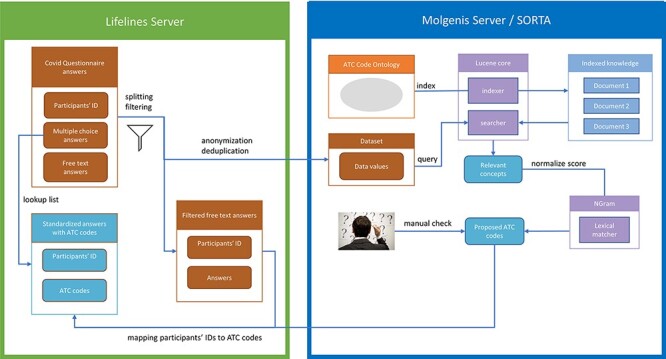
Process overview. Pre-processing and anonymization occurs on the Lifelines server. Actual mapping with SORTA takes place on the Molgenis server. Finally, the results are mapped back using the pre-processed answers. Adapted from Pang et al., 2015 (9).

As it is a standard operating procedure to anonymize Lifelines data when processing them outside of the Lifelines server, we pre-process the data to create two tables. The first table contains participant IDs and their respective answers. The second table contains only the anonymous and deduplicated answers without the participant IDs. We then load the anonymized answers into Molgenis for semi-automatic mapping. The SORTA results contain the Unique Resource Identifiers (URIs) of the matched ontology terms, for which the ATC codes are known. Thus, adding the ATC codes is just a lookup task that can be automatically performed. However, before the results are sent back to the Lifelines server, terms flagged for review within SORTA need to be manually curated. Once this is carried out and the data uploaded back onto the Lifelines server, the answers with ATC codes are recombined with the participant IDs using the table with participant IDs as a lookup list. Figure 2 shows an overview of the full process.

### Pre-processing the free-text input

The Lifelines COVID-19 questionnaire data are reported as tables with participants as rows and questions as columns. For our purpose, only the medication-related questions COVID24A2–COVID24A10 were relevant (see Supplementary Material Attachment 5 for a complete list of these questions). These questions contained both multiple-choice and free-text responses, with the first eight questions related to specific pharmaceutical categories and the last question allowing the user to list any other drugs that did not fit into the previous categories. While the ATC codes for the predefined drugs are known, the free-text responses need further processing. We could have uploaded the answers to SORTA directly but decided to do some automated pre-processing to speed up the mapping process.

In the first pre-processing step, we converted the text to lower case. Next, we added missing spaces after commas. We then performed removal/escaping of special and escape characters (tabs, quotes, brackets, semicolons and ‘—’) to avoid unwanted interaction with our code. We further used a corpus of Dutch stop words from the Natural Language Toolkit (https://www.nltk.org/) to eliminate common stop words and defined a list of terms to be removed that included common domain-specific descriptions and filler words (like ‘elke dag’, ‘plus’ and ‘forte’) and the most common pharmaceutical company names. The transformation into lower case and removal of certain words were performed mainly to deduplicate entries. This allows us to combine terms with the same spelling mistakes so that the expert user only needs to check each term once. Since drug dosage does not play a role in determining the ATC codes, we removed all information about weight, volume or concentration (e.g. ‘5 gram’, ‘100 ml’, and ‘1 g/ml’) using regular expressions.

We further recognized that splitting the text into individual words or word combinations would help in dealing with cases where several drugs were input in one field. Without splitting the answers into parts, SORTA would suggest a list of the 10 most likely drugs with the highest score, which in many cases would be the longest word due to the N-gram-based mapping algorithm. We defined some exceptions for terms that belong together, like ‘vitamin c’. The form of application of a drug also sometimes plays a role in the ATC code classification, so we had to define exceptions for those cases to keep the drug and application form together (e.g. tablet, nasal spray and cream). We also removed unnecessary spaces and codes for empty answers (e.g. 9999 or 8888). Any text fragments with fewer than three letters remaining after splitting the answer were discarded. The Python scripts used for the pre-processing are available on GitHub (https://github.com/AJKellmann/Scripts-for-the-translation-of-medicine-usage-data-to-ATC-codes). Subsequent cleaning steps removed special characters, multiple white spaces, empty strings, words with fewer than three letters and duplicates. Finally, the complete, filtered but not split answer was added as a ‘Synonym’ to each of the separated terms. Synonym is a keyword for SORTA. SORTA will take Synonyms into account for the mapping and display them beneath the term they belong to so that the human curator can also see the context. Depending on how clean the initial data were, several of the pre-processing steps could be skipped. However, additional steps like an automatic spelling correction might be helpful in future applications.

### Creating a Dutch ATC code ontology

Good, well-curated ontologies exist for languages spoken by large numbers of people in multiple countries, e.g. multiple such ontologies exist for English. For languages like Dutch that are spoken by fewer people, developers may need to build their own ontologies by curating, cleaning and combining existing resources. We built our Dutch ATC ontology, which connects the ATC code hierarchy with the Dutch drug names in several steps, by integrating information from multiple databases. We used the ATC classification ontology of the WHO from BioPortal (https://bioportal.bioontology.org/ontologies/ATC) as a basis because it contains the ATC code hierarchy as RDF/ttl format. Within SORTA, only the classes from an ontology are mapped to the input terms. Therefore, we encoded the relationship between ATC codes and drugs as a hierarchical class–subclass relationship (see Supplementary Material Attachment 4), which allows SORTA to check for the drug names and the names of the ATC code groups.

To obtain the relationship between the Dutch drug names and their ATC codes, we first considered the G-Standaard, a rich drug database maintained by Z-Index (https://www.z-index.nl). To access the G-Standaard, we requested a database excerpt with the relevant drug names and ATC codes from the IADB.nl database (http://www.iadb.nl/) (11). This excerpt contained about 177 387 names of medical products, including both drug names and many other non-medication items, distributed by participating pharmacies. Only 28 338 of the listed items were Dutch drug names with related ATC codes, and some entries contained not only the name of the drug but also the form of application, abbreviations, concentrations, dosage information or company names. We therefore performed some data cleaning before adding the data into our ontology, e.g. we removed duplicate entries that reflected differences in package size (see Supplementary Material Attachment 2 for examples of data before and after cleaning). After the data cleaning, this added 8900 distinct pairs of drug names and ATC codes to our ontology.

On the website of the Dutch Foundation for Pharmaceutical Statistics [Stichting Farmaceutische Kengetallen, (SFK)] (https://www2.sfk.nl/producten/classificatie_index/atcboom/), we found a very clean database of relations between drug names and ATC codes. Here, it was possible to get information about Dutch drugs and their related ATC code(s) based on either the name of the drug or a (partial) ATC code. Compared to IADB.nl, the SFK data did not contain abbreviations or package sizes and therefore needed less data cleaning (see Supplementary Material Attachment 1 for examples from before and after data cleaning). We thus ended up with about 3700 distinct drug–ATC code relations and merged them into our ontology.

We also checked the Dutch version of DBpedia for their data schema for drugs and ATC codes (http://nl.dbpedia.org/web/). DBpedia (https://dbpedia.org/org/) contains computer-readable data extracted from Wikipedia in the form of Linked Data. The information provided about the drugs includes not only ATC codes but also information like other identifiers or brand names. Reuse of existing URIs whenever possible is good practice when working with Linked Data to keep it interoperable. Therefore, it was interesting to see which predicates DBpedia uses to encode ATC codes. We used the following SPARQL query to query for the relevant data from the SPARQL end-point at http://nl.dbpedia.org/sparql/ (26 May 2020):

select distinct ?a as ?Resource str(?label) as ?Name

?a as ?Resource str(?label) as ?Name

CONCAT(str(?prefix), str(?suffix)) as ?ATC

CONCAT(”http://purl.bioontology.org/ontology/UATC/”, str(?prefix), str(?suffix)) as ?URI

str(?merknamen) as ?Merknamen

where {

?a <http://nl.dbpedia.org/property/atcPrefix> ?prefix.

?a rdf:type dbpedia-owl:Drug.

OPTIONAL {?a rdfs:label ?label}

OPTIONAL {?a <http://nl.dbpedia.org/property/atcSuffix> ?suffix.}

OPTIONAL {?a <http://nl.dbpedia.org/property/merknamen> ?merknamen.}

}

The result contained 517 different drugs with several brand names (‘*merknamen*’) and ATC codes, of which 454 had valid ATC codes. Invalid entries did not match the expected ATC code format (e.g. ‘9.0CA04’). To ensure that the ATC codes of the 454 drugs were annotated correctly, we compared the results to our previous sources. Of the 454 entries, 326 were already in our collection with the same ATC code, while 121 entries were not included in the other sources. We verified these new entries using the official website of the WHO Collaborating Centre for Drug Statistics Methodology (https://www.whocc.no/use_of_atc_ddd/) before adding them to our ontology (see Supplementary Material Attachment 3 for examples). Of these 121 entries, 113 turned were valid and 8 had to be corrected. We also corrected those entries in the Dutch DBpedia.

### Mapping free text to ATC codes using SORTA

This section describes how to carry out mapping within SORTA.

#### Uploading the ontology and input file into SORTA

For the mapping process, SORTA requires an ontology corresponding to the terms that need to be matched and a semicolon-separated text file with the terms that need to be matched to the ontology terms. For our use case, we uploaded the ontology described earlier, which contains a hierarchy of ATC codes as classes and drug names as subclasses. The input file can either be uploaded as a text file or pasted as text into the website. SORTA uses some keywords like ‘Name’ and ‘Synonym’ to determine which terms should be mapped to the ontology. Uploading and processing the file, especially with thousands of terms, can take several minutes. However, it is not necessary to wait for the file to be processed entirely before starting the next SORTA job.

#### The semi-automatic mapping

At this point, SORTA has performed an automatic mapping based on the similarity of the input terms (in this case, the drug name) to the drug names with known ATC codes from the ontology. In the best case, a mapping is an exact match, and no manual interaction is necessary. This was the case for 30–40% of the Lifelines COVID-19 questionnaire free-text results. For the remaining terms, SORTA uses Apache Lucene to attempt to find the best match based on the term itself and the Synonyms and provides a similarity score based on an N-gram-based algorithm for a given uniform cut-off value (9). A word that contains a spelling mistake with, e.g. one letter replaced, will be mapped with a high confidence value. Users can define the threshold to which slight differences will still be automatically accepted. Terms with a lower confidence value need to be confirmed (‘Match’) or declined (‘No Match’) by the user. SORTA therefore provides a list of suggestions with the highest confidence values for each input term. For cases where the expert does not agree with any of the terms SORTA suggests and thinks that there might be another solution, the term can be flagged for review and curated manually after downloading the results.

**Figure 3.**
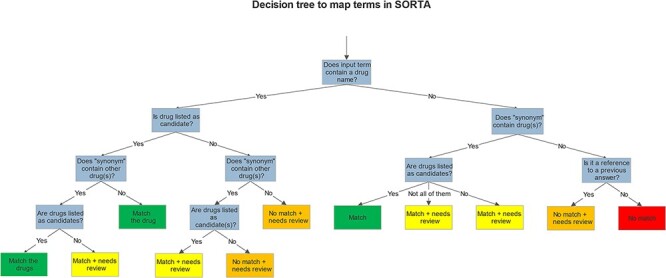
Decision tree shows how we mapped and flagged terms in SORTA.

While SORTA was initially designed to make a 1:1 mapping between input and ontology terms, our mapping task required multiple mappings to account for the possibility of multiple drugs listed in one answer. However, since we pre-processed the input terms, multiple drugs per entry rarely occurred. To retain useful context for the user, we also kept the whole answer as a SORTA ‘Synonym’. Having multiple drugs as Synonyms made it possible to map multiple drugs per answer for a selected term. It is then up to the user to decide whether to take only the term itself or all the drugs mentioned within the Synonym field into account for the mapping. Both choices are acceptable; taking the Synonyms into account may lead to redundancy within the SORTA results. We decided that this redundancy was acceptable and could be handled automatically during the mapping to ATC codes since our end goal was to find all ATC codes matching each participant’s full answer. Therefore, we agreed on the scheme for mapping shown in the decision tree in Figure 3.

After mapping, we downloaded the results, matched the ontology terms to their related ATC codes with a script and grouped the terms marked for review by the ‘Synonym’. We performed manual curation using Excel. Grouping the answers by the ‘Synonym’ (the slightly filtered version of the complete answer) made it easy to review whether all the drug names in an answer had been annotated with ATC codes. Each group that contains at least one term marked for review had to be checked. The manually curated answers are then sent back to the Lifelines server. A screencap of this manual curation step is shown in Table 1.

**Table 1.** Manual curation step in Microsoft Excel

Name	Synonym	ontologyTermName	score	validated	review	Atccode	Atccode text
ace	ace remmer		0.0	True	True	nan	
omeprazol	acenocoumarol/omeprazol/losartan kalum/lercanidipine	omeprazole	100.0	False	False	A02BC01	omeprazole
acenocoumarol	acenocoumarol/omeprazol/losartan kalum/lercanidipine	acenocoumarol	100.0	False	False	B01AA07	acenocoumarol
lercanidipine	acenocoumarol/omeprazol/losartan kalum/lercanidipine	lercanidipine	100.0	False	False	C08CA13	lercanidipine
losartan	acenocoumarol/omeprazol/losartan kalum/lercanidipine	losartan	100.0	False	False	C09CA01	losartan
kalum	acenocoumarol/omeprazol/losartan kalum/lercanidipine	acenocoumarolum	47.06	True	True	B01AA07	acenocoumarol
kalum	acenocoumarol/omeprazol/losartan kalum/lercanidipine	lercanidipine hcl 20 mg t om	43.04	True	True	C08CA13	lercanidipine
thoraxen	acetyllsalicyzuurcardio, thoraxen	acetylsalic cardio	60.0	True	True	B01AC06	acetylsalicylic acid
acetyllsalicyzuurcardio	acetyllsalicyzuurcardio, thoraxen	acetylsalic cardio	73.17	True	True	B01AC06	acetylsalicylic acid
perindopril	acetylsalicylzuur cardio/perindopril	perindopril	100.0	False	False	C09AA04	perindopril
acetylsalicylzuur	acetylsalicylzuur cardio/perindopril	acetylsalicylzuur	100.0	False	False	N02BA01, B01AC06	acetylsalicylic acid
cardio	acetylsalicylzuur cardio/perindopril	acetylsalicylz cardio	71.19	True	True	B01AC06	acetylsalicylic acid
acetylsalicylzuur dispertablet	acetylsalicylzuur dispertablet	acetylsalicylzuur	73.47	True	True	N02BA01, B01AC06	acetylsalicylic acid
acetylsalicylzuur dispertablet	acetylsalicylzuur dispertablet	dispertablet	59.09	True	True	Y	

The ‘Synonym’ column shows a slightly filtered version of the participant’s complete answer. The ‘review’ column indicates which terms need to be checked manually. The terms are grouped by the ‘Synonym’ column to display all parts that belong to the same answer simultaneously.

## Results

The Lifelines COVID-19 surveys were sent out weekly between 28 March 2020 and 18 May 2020 and biweekly thereafter. During the first week, 42 539 Lifelines participants took part in the survey. For the nine free-text questions, we received 25 276 replies in just that first week. To enable SORTA to support experts in mapping replies to ATC codes, we built an ontology containing most Dutch drug names and their respective ATC codes, described earlier. We also worked on the SORTA interface and wrote scripts for pre-processing reducing the number of terms that needed to be curated by anonymization and removing duplicates (this led to a reduction of a factor of ∼2.3 for the first questionnaire) and post-processing of SORTA results. The questionnaire was then optimized to further reduce the number of terms needing manual evaluation by adding a question about whether a participant’s medication usage had changed since they last filled out the survey. If so, the participant was asked to fill out their information again. This reduced the number of free-text answers to ∼10% for follow-up time points. All these steps reduced the number of free-text answers that needed to be processed manually and speed up the mapping process. On average, we matched 2.56 answers (3125 terms) per minute to ATC codes during the manual curation with Excel and 5.25 answers (6119 terms) per minute with SORTA and the subsequent curation step. Therefore, we were twice as fast with the semi-automatic method. The time-savings are largely due to (i) the cases that are solved automatically and (ii) matching the ATC codes automatically, so the human user only needs to match the given answer to the ontology term and does not need to look up the ATC code manually.

### User interface enhancements

**Figure 4.**
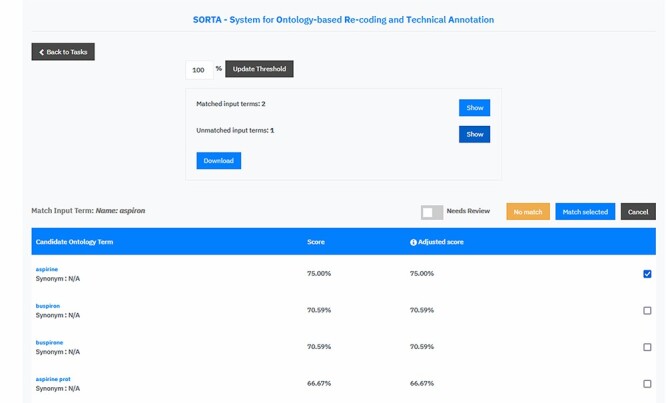
Screen capture of the SORTA 2.0 user interface. In contrast to the original version of SORTA, the SORTA 2.0 user interface allows users to map multiple terms at once and to flag a term for a manual review.

We extended SORTA to be able to flag terms for curation and to map more than one drug name with a known ATC code(s) to an input free-text string (see Figure 4 for a screenshot of the updated SORTA user interface). The first option in the SORTA Web interface is to update the threshold for the cut-off value (set to 100% in the screenshot). This determines up to what level of algorithm–confidence SORTA suggestions should be counted as matched terms. Entering a threshold >100% will lead to a situation where all terms are shown as unmatched terms. For terms with confidence values below the user-defined threshold, SORTA suggests the most likely matching terms, so an expert can quickly decide whether one of the suggestions was correct. This allows an expert to concentrate on the more complex cases and perform the mapping task much faster.

Initially, SORTA was designed for a 1:1 mapping, with one input term mapped to one ontology term. However, we soon realized that some COVID-19 questionnaire answers listed multiple drugs in one line, which resulted in multiple suggestions mapping to different parts of the answer. To help resolve this issue, we adapted SORTA by adding the option to map multiple terms at once. In addition, we replaced the option to choose one ontology term with a list of check boxes and allowed multiple results with the ‘Match selected’ button.

Another new feature is the toggle button ‘Needs Review’. This was added to flag input terms for a manual review, e.g. when SORTA does not suggest the correct term as an option. The ‘Needs Review’ flagging can be combined with the ‘No match’ and the ‘Match selected’ option.

### Evaluation in the Lifelines COVID-19 dataset

To evaluate the methodology, we mapped the answers of the first three questions of the first week of COVID-19 questionnaires to their ATC codes (see Table 2) shows an overview of how many of the 42 539 participants answered each of the medication-related questions^1^ in the first week. To reduce the amount of manual curation, we deduplicated the results and combined identical answers. The more replies we got within one category, the higher the effect of the deduplication, as multiple participants input the same drug names and common spelling mistakes were repeated. The exact numbers can be found in Table 2^2^. However, many of those answers consisted of multiple drugs or at least multiple words. SORTA would treat these as one term, resulting in a poor mapping score. We therefore separated those and pre-processed them independently. We also kept a (slightly filtered) version of the free-text answer with each term so that the reviewer and SORTA would retain the context. This not only increases the number of individual terms to evaluate (as shown in Table 2^3^) but also helps SORTA find better mappings and ensures that all terms are addressed for multiple drugs listed in one line.

**Table 2.** Number of free-text entries recoded. It contains only the results of Week 1. Only the first three questions have been processed by an expert using SORTA

Question	COVID24A 2	COVID24A 3	COVID24A 4	COVID24A 5	COVID24A 6	COVID24A 7	COVID24A 8	COVID24A 9	COVID24A 10
Number of responses^1^	4320	827	79	874	761	258	237	1247	16 673
Number of responses after deduplication^2^	1627	435	65	663	514	166	174	735	6662
Number of terms extracted from these files, used as input^3^	1834	640	74	1040	689	214	279	971	7267
Automatic match (100% confidence)^4^	829	257	45	419	265	105	77	422	2569
Automatic match (83% confidence)^5^	1106	304	51	484	353	121	96	495	3561
Manual mappings created within SORTA^6^	868	317	27						
Values marked for manual reviewed in SORTA results^7^	211	62	2						
Number of pairs of ‘input term and free-text answer and ATC code’ after manual curation (Multiple ATC codes per drug and multiple terms per input possible) ^8^	1842	586	93						
Number of unique pairs of ‘free-text answer and ATC code’ after manual curation (Multiple ATC codes per drug and multiple terms per input possible)^9^	1575	500	87						
Number of unique pairs of ‘Participant and ATC code’ (Multiple ATC codes per drug and multiple drugs per answer possible)^10^	4885	994	117						

After loading the terms into SORTA, it performs an automatic mapping to the ontology. The threshold is the cut-off value for accepting automatic mappings. The default value is 100%, which means that only input terms that are the same as in the ontology are mapped^4^. Lowering the threshold of the confidence value means accepting words that might have slight differences. We checked the terms manually and found the first mismatches for the highest-ranked SORTA suggestion ∼83% threshold^5^. Most of the remaining cases could be solved within SORTA^6^. However, when SORTA did not suggest the correct answer, we flagged the term for a manual review. SORTA also sometimes suggested terms based on additional drug names from the ‘Synonym’, which could still consist of multiple drugs. These were then handled during the manual curation step^7^.

After downloading the results from SORTA, we combined the mapped terms with the ATC codes in our ontology. This is an n:m mapping since there are cases where one drug has multiple ATC codes and where different spellings of the same drug have the same ATC code(s). Even so, we discarded some input terms within SORTA as the number of entries seems to be bigger than the number of terms, we uploaded into Molgenis^8^.

If different terms of the same answer (‘Synonym’) have been mapped to the same ontology terms, it is possible to group them to combine duplicate entries^9^. The last step is to recombine the ATC codes with the participants’ IDs^10^. This step reverses the reduction of terms that we initially achieved by deduplication. With the exception of a few answers that an expert rejected (e.g. ‘Can’, ‘Android’ or ‘Hydro’), all drugs were mapped to ATC codes.

**Figure 5.**
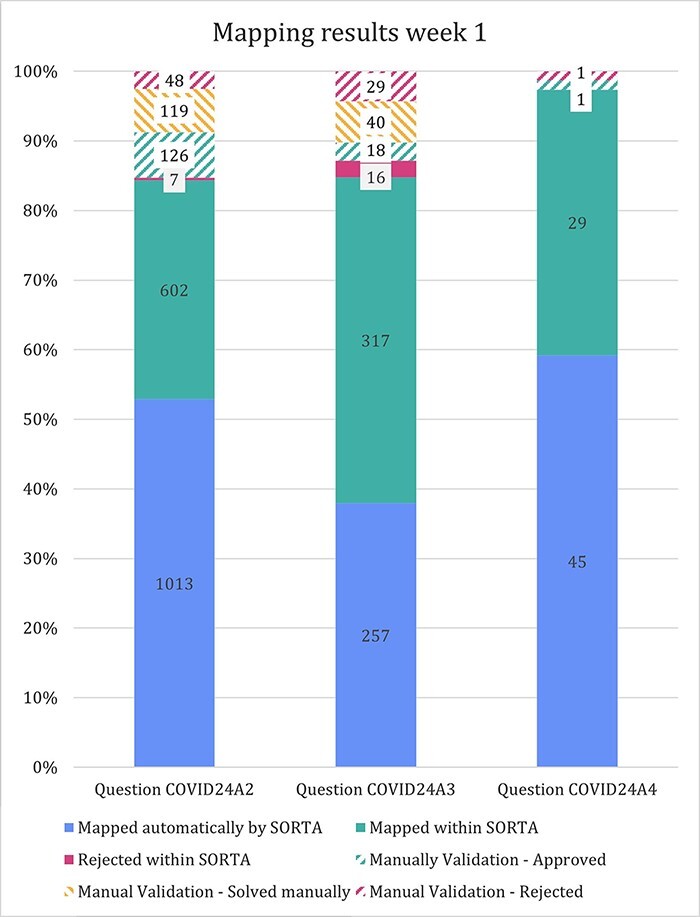
Stacked bar diagram showing the input terms of the first three free-text questions for the first week of Lifeline’s COVID-19 questionnaire responses.

The stacked bar diagram in Figure 5 shows how many terms were mapped automatically and how many cases could (or could not) be solved within SORTA and during the manual curation step. For question COVID24A3, we confirmed all terms manually (threshold of 100%). For questions COVID24A2 and COVID24A4, we used a cut-off value of 83% confidence before starting the manual mapping process.

In the previous version of SORTA, it was not possible to map one input term to multiple ontology terms. However, we found it helpful when there were multiple different drugs or valid options in one answer. For the first free-text question (COVID24A2), we loaded 1834 terms into SORTA. The number of terms in the output (including rejected terms) was 1913, and the number of terms actually increased to 1915 during manual curation. This was unexpected as we had pre-processed the answers and split them into parts. The two additional entries were caused by a missing space between two drugs (‘spironolactionomeprazol’ and ‘cholecalciferolpantaprasol’) that we had to split up manually.

### Assessing the performance of the semi-automatic mapping

We compared our method to human curation to assess time-savings and performance. For this comparison, a dataset of 100 terms that was also pre-processed was manually curated in Microsoft Excel. It is important to note that the pre-processing step reduces the number of terms to be curated. Since the total pre-processing reduction will vary substantially between datasets, we have excluded this gain from our comparison. For optimal comparison, both curations were performed by the same person.

The fact that the updated version of SORTA allows multiple answers for one input term complicates the calculations for assessing performance. It is possible to take the human-curated terms and ask SORTA to annotate them. In this case, we will receive the first best result. Precision, recall and F-score can then be calculated for different thresholds based on human-curated output as a gold-standard set. There are two options for how we handle input terms that were and should be matched to multiple terms:

- We consider SORTA successful if one of the input terms was found. In a case of multiple mappings by the human curator, duplicate inputs are removed. This results in an overestimation of the F-scores.
- We compare each output value with SORTA’s values and penalize the missing values by treating them as mismatches (false positives). SORTA will always show the same output for the same input term. Therefore, it will fail in all cases with multiple results. This results in an underestimation of the F-scores.

**Figure 6.**
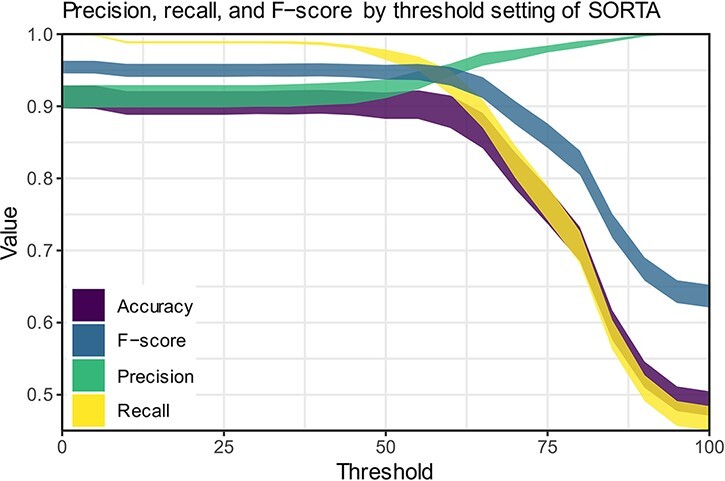
Precision, recall and F-scores for different threshold settings within SORTA. (Based on the automatic mapping of the answers for question COVID24A2 by SORTA compared to manual curation).

Both options have their opposite limitations, and we can view them as upper and lower limit estimates for the performance of our method. We have therefore calculated precision, recall and F-score for both scenarios (see Table 3) and calculated each for thresholds within SORTA ranging from 0 to 100 (see Figure 6).

**Table 3.** Statistic formulas

$Accuracy = \frac{{TP\, + {\rm{\,TN}}}}{{{\rm{TP}} + {\rm{TN}} + {\rm{FP}} + {\rm{FN}}}}$	(1)
$Precsision = \frac{{TP}}{{TP + FP}}$	(2)
$Recall = \,\frac{{TP}}{{TP + FN}}$	(3)
$F-score = 2*\frac{{Precision\,*\,Recall}}{{Precision\,+\,Recall}}$	(4)

TP, true positive; TN, true negative; FP, false positive; FN, false negative.

## Discussion

We built a system within Molgenis SORTA to support pharmacology experts in translating Dutch drug names in free-text form into standard ATC codes. By solving a large proportion of the cases automatically (27–61% with 100% confidence and 34–69% with 83% confidence), SORTA reduces manual work to a fraction. For most of the remaining terms, SORTA suggested the correct solution so that the number of cases that could be solved within SORTA was 84–97%. Additionally, mapping drug names to drug name suggestions is still faster than manually looking up the ATC codes. Only the most difficult cases need to be solved outside of SORTA, and we assume that mapping these difficult cases to ATC codes takes as much time as it would cost when solving these particular cases entirely manually.

In previous Lifelines studies, the matching of Dutch drug names to ATC codes was done solely manually to prevent mismatching ATC codes. This was mainly for two reasons. On the one hand, the researchers wanted to be sure that the ATC codes were correct instead of relying on a black-box machine learning algorithm that still accepts a small percentage of errors. On the other hand, there was a lack of training datasets based on Dutch drug names like those that exist for the English language.

Previous approaches for drug name entity recognition have been established to recognize drug names in medical texts. Tang *et al.* present a general best practice approach for drug name recognition (5). Sanchez-Cisneros *et al.* presented a version where they use ontologies and data dictionaries, as we do in our approach (6). Particularly for information extraction from free-text questionnaires, Ramachandran provides valuable insights in his thesis (4). Current machine learning approaches can already identify and pre-process drug names quickly and with high accuracy in free texts (12). These programmes mostly use training datasets that exist for English drug names (e.g. extracted from Drugbank or PubMed). However, we did not have such a training dataset for Dutch drug names and had to take potential spelling mistakes into account. Therefore, we built a dataset in the form of an ontology. This ontology and the results from the human data curation (especially the negative examples) can be used as part of a training dataset with Dutch drug names.

### One possible future solution: autocompleting participant free-text answers (but is the underlying database complete enough?)

Mapping human-entered medication data to a codified ATC data format commonly involves much manual labour. Our method already reduces this effort by half and even more when including pre-processing, but there are other ways to further speed up this process. One obvious way to avoid manual mapping would be to ensure that Lifelines participants fill in complete medicine details themselves, which could be assisted by autosuggest or autocomplete functions. The participant knows best which drugs they are taking, making them the best curator, and this would have the added benefit that participants would be able to complete the survey faster, which might increase willingness to fill out future surveys. As prospective biobanks like Lifelines depend heavily on participant involvement, this could potentially increase the value of the biobank.

Use of an autosuggest/autocomplete function requires a list of options to be displayed to the participant, so one would require such a function in the online survey software. This could be handled using browser-based scripts, optionally in combination with server-side APIs. Having an autosuggest/autocomplete function also requires a set of words (in this case, drug names) as options. Even if the function contained just a selection of the most frequently used drugs, this would prevent many misspellings. However, a completely closed selection of drugs might not work. While the G-Standaard contains a complete list of all prescription drugs on the Dutch market, it does not cover all possible drug names and ways of spelling them. There are still drugs with different separators or word orders that may not be covered, e.g. drugs imported from abroad. Moreover, not all over-the-counter drugs or pharmaceutical products are in the G-Standaard, especially for those sold outside of pharmacies.

### Further improvements of our method

There are several ways to further improve our method. During the pre-processing step, we used regular expressions to strip dosage information and concentrations from the free-text input. While this worked in many cases, using regular expressions on free-text input may lead to errors and unexpected results. For example, within the 2548 terms we processed, there were two cases where a term was accidentally shortened, thereby changing the participant’s answer. Another issue that could already be resolved during pre-processing is the handling of misspelt terms. One way to handle misspelt terms would be to use a spell checker, but in some cases, this also changes the participant’s answer. In both cases, we need to consider whether we want to keep the participants’ original answers or to use such tools to reduce manual curation while accepting the possible introduction of errors. Furthermore, dosage information could also be queried in a separate field in the online survey software. While this would make filling out the survey more time-consuming, it would help in obtaining cleaner data. A separate dosage field would also enable the calculation of prescribed daily dose information, which in turn opens up new research opportunities.

While working with the free-text medication data, we soon noticed that some spelling mistakes or abbreviations were repeated by different participants. We dealt with this by deduplication during pre-processing, but we could take another approach. If we added the different ways to (mis-)spell a drug name to the ontology, SORTA could automatically recognize them the next time. However, adding misspelt terms to the ontology might lead to mix-ups between the correct and misspelt terms. Therefore, we need to think of a way to reduce the risk of introducing errors while maintaining the benefit of a self-learning ontology. Currently, SORTA only accepts the subclass relationship as well as Synonyms. To prevent confusion between correctly spelt and misspelt terms, predicates like ‘skos:hiddenLabel’ (https://www.w3.org/TR/skos-reference/#labels) might help. Unfortunately, such predicates are not yet supported in SORTA and would need further development. It may also be possible to add additional information (like brand name) to the drug names in the ontology and include those into the mapping process.

Furthermore, SORTA currently accepts all letters from a to z and A to Z and numbers 0–9 and converts them to small letters for comparison (9). However, letters with diacritics such as the Dutch ‘ï’ are not supported, and this can affect drug names that contain that letter, e.g. ‘Tretinoïne’. For Dutch, the consequences of the characters SORTA accepts are limited, but for languages that use a different alphabet (for example, Greek) or writing system (for example, Chinese), the SORTA approach is unsuitable.

During the manual curation, we sorted and grouped inputs based on Synonyms to assist the expert in executing the task. We then recognized that sorting and grouping inputs by Synonym instead of by confidence within the SORTA user interface could have also helped the expert. For example, grouping by Synonym would allow us to group the different parts of the same answer. Grouping by the most likely mapping could also be helpful. A curator could then do the mapping of terms that belong to the same question or the same drug in one go.

### Limitations of our Dutch ATC ontology

**Figure 7.**
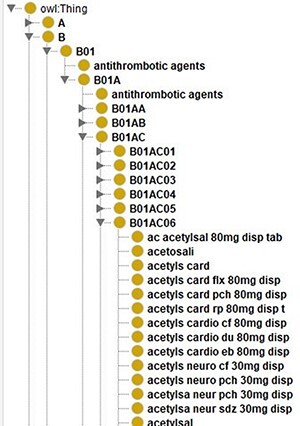
Screenshot of the class hierarchy of the ATC code ontology in Protégé (13). It shows that different names of aspirin as leaves of the ATC code hierarchy’s tree structure.

For this project, we created an ontology that contains Dutch drug names related to their ATC codes as shown in Figure 7. To create an ontology, you need to represent each resource using a URI. Here, we just used a prefix (‘http://www.UMCG.nl/’) and added an MD5 hash value of the drug’s label. Interoperability with existing ontologies or resolving the URIs was deemed irrelevant since it is merely part of an intermediate step.

Currently, the ontology contains all the different ways to spell a drug as separate classes. In the case of aspirin, there are 140 different classes. Instead of treating them separately, we could use the predicates that SORTA uses to encode Synonyms and encode these different spellings of one drug as one class. When keeping them separate, SORTA might suggest multiple forms of the same drug as possible matching results. In the case of multiple drugs per line, this can lead to SORTA listing the same ATC code multiple times while other drugs are not in the top-10 suggested terms. When we encoded all the terms that encode the same ATC code as Synonyms using the URI ‘http://www.ebi.ac.uk/efo/alternative_term’ as predicate, SORTA only listed the ATC code related to these entries once. When we compared the results of both versions with the manually annotated values for a small sample of data, we realized that the results had also gotten worse. Only 37 (62%) rather than 48 (80%) of the 60 terms were still correctly mapped when we combined the terms. This also led to about a 5-fold increase in the amount of memory SORTA uses and in the time it takes to process the data.

### Impact

We estimate that it took roughly 960 h to develop this method. This estimation includes writing the scripts for pre- and post-processing, extending SORTA, data selection and preparation to build the ontology and curation of some of the data. Since using SORTA doubles the processing speed for the mapping of ATC codes, it will pay off (in terms of time) after the amount of time we spent on building it. When processing 180 answers per hour for human curation and 360 answers per hour with our SORTA method, the time investment would be repaid after 176k answers. This does not include the effect of pre-processing (anonymization, deduplication, etc.). The effect of removing duplicates reduced the number of answers to be curated by another factor 2.3 for the first questionnaire. The effect of pre-processing varies depending on the fraction of duplicate answers. If we assume this 2.3-fold reduction by pre-processing, the whole method would be repaid after about 70–80k answers. To put this into perspective: the first two rounds of questionnaires for the Lifelines COVID-19 Cohort had ∼25k answers containing drug names each. In subsequent questionnaires, only changes in drug use had to be reported, which reduces the number of answers to ∼3k answers per questionnaire. With these numbers, it is possible that we earned back our time investment solely when applying it to this cohort.

Our method can be reused for future mapping of drug names to standard ATC codes, for example, in processing future survey results in Dutch biobanks like the Lifelines Cohort Study. In addition, it is possible to reuse and adapt our ontology. We also learned how to better ask open questions (i.e. asking participants whether their drug usage has changed, adding autocomplete functionality). In addition, offering ATC codes as a result instead of free text facilitates further analysis of the data. For example, it would be possible to query groups of participants who take specific therapeutic subgroups, e.g. antihypertensive drugs (ATC code C02). Furthermore, when combining drug use with other participant information, clinical phenotypes could be determined with higher confidence. Our method thus facilitates having high-quality medication data available within biobanks without the need to link to drug prescription or dispensing databases while still maintaining sufficient sample sizes.

In addition to using the method solely for drug names that need to be matched to ATC codes, the method can also be applied to other use cases and ontologies. Examples of these can be found in the original SORTA paper, where SORTA was used for encoding different types of sports with the Metabolic Equivalence of Task ontology and for encoding phenotypes with the Human Phenotype Ontology (9).

When applied to a new domain, the main work will be the curation (or downloading) of a new ontology plus, potentially, various term definitions. Most of the time will be spent on finding relevant sources and potential data cleaning. A new ontology can also be created by manually curating a pilot set or by sourcing existing dictionaries of relevant synonym–term mappings. During the curation process, the ontology can be updated with the human-curated mappings to improve the accuracy of SORTA. Once we knew which data sources to use for our ontology, we created it within 3 weeks because we could already source from dictionaries and an existing human-mapping task. Building a new ontology will require some knowledge of the domain but will not require in-depth expertise (i.e. the ontology engineer in this project had a medium level of expertise on the domain subject). Once an ontology exists, we recommend using SORTA if more than a couple of 100 terms need to be annotated.

## Conclusion

We adapted Molgenis SORTA to match answers from the Lifelines COVID-19 questionnaire to ATC codes. To do so, we created an ontology containing the relation between Dutch drug names and their respective ATC codes. We then enhanced SORTA by adding the option to map to multiple ontology terms at once and to flag terms for a manual review. On average, the revised version of SORTA automatically matched 44.6% of the results to ATC codes at a threshold of 100%. The first mismatch of SORTA’s highest-ranked suggestion occurred at ∼83% confidence threshold. Using this threshold, SORTA could, on average, match 58.8% of the terms correctly and automatically. Although most cases could be solved within SORTA, about 10–15% were flagged for review and about 5% had to be solved manually by an expert outside of SORTA. With about 40–50k completed questionnaires being returned for each questionnaire round, the total amount of data to be curated was high. Although SORTA does more than half of the mapping automatically and significantly speeds up the manual curation process, the total number of replies was large and therefore still quite time-consuming to process. In total, we spent about 7 h (412 min) mapping 85–95% of the 5226 free-text answers of the first three COVID-19 questionnaire questions of Week 1 to ATC codes in SORTA.

Particularly, for large amounts of free-text answers, we recommend removing duplicates before uploading them into SORTA. Even so, the pre-processing was quite effective. Our most effective reduction in answers to curate came with changing the Lifelines questionnaire to ask only for drug names when usage had changed. This results in less effort for the expert and saves participants’ time when filling out the survey. Whether and how much pre-processing of the data is required depends primarily on how many replies there are. At minimum, we recommend removing duplicates and empty fields.

If we were to use this method for additional surveys in the future, we would ask for the drug name and optionally the brand in separate fields. We would also include a separate field for dosage information. We can also build upon our current version of the Dutch ATC code ontology and iteratively improve it by adding previously curated terms.

The data that we have curated for the Lifelines COVID-19 Cohort has been given back to Lifelines to make it available to other researchers. To the best of our knowledge, it has not yet been used in subsequent analyses. However, our method and scripts were successfully adopted to process similar surveys within the Lifelines NEXT birth cohort and were greatly appreciated (14). In that questionnaire, our recommendations to ask for drugs, dosages and the brand name separately were followed. Having these relatively clean answers meant that automatic data cleaning pre-processing could be skipped, and the answers were mapped to terms from our ontology with SORTA. Afterwards, our scripts were used for the post-processing to fully automatically translate those ontology terms back to ATC codes. We received the feedback that our method has saved a lot of time and it will be used again in the future.

## Supplementary Material

baad019_Supp

## Data Availability

The data analysed in this study were obtained from the Lifelines Biobank under project application number ov20_0554. Requests to access this dataset should be directed to the Lifelines Research Office (research@Lifelines.nl). The scripts we wrote as well as the ontology we created are available at https://github.com/AJKellmann/Scripts-for-the-translation-of-medicine-usage-data-to-ATC-codes. The Molgenis Plugin SORTA is part of Molgenis, which can be found here: https://github.com/molgenis/molgenis.

## Lifelines Corona Research Initiative

We acknowledge funding for the Lifelines Corona Research project from the University of Groningen and the University Medical Centre Groningen. The authors wish to acknowledge the efforts of the Lifelines Corona Research Initiative and the following initiative participants:

H. Marike Boezen^1^, Jochen O. Mierau^2,3^, Lude H. Franke^4^, Jackie Dekens^4,5^, Patrick Deelen^4^, Pauline Lanting^4^, Judith M. Vonk^1^, Ilja Nolte^1^, Anil P.S. Ori ^4,6^, Annique Claringbould^4^, Floranne Boulogne^4^, Marjolein X.L. Dijkema^4^, Henry H. Wiersma^4^, C.A. Robert Warmerdam ^4^, Soesma A. Jankipersadsing^4^, Irene V. van Blokland^4,7^

^1^Department of Epidemiology, University of Groningen, University Medical Center Groningen, Groningen, The Netherlands

^2^Faculty of Economics and Business, University of Groningen, Groningen, The Netherlands

^3^Aletta Jacobs School of Public Health, Groningen, The Netherlands

^4^Department of Genetics, University of Groningen, University Medical Center Groningen, Groningen, The Netherlands

^5^Center of Development and Innovation, University of Groningen, University Medical Center Groningen, Groningen, The Netherlands

^6^Department of Psychiatry, University of Groningen, University Medical Center Groningen, Groningen, The Netherlands

^7^Department of Cardiology, University of Groningen, University Medical Center Groningen, Groningen, The Netherlands

## Lifelines Cohort Study

The Lifelines Biobank Initiative has been made possible by funding from the Dutch Ministry of Health, Welfare and Sport; the Dutch Ministry of Economic Affairs; the UMCG, the Netherlands; the University of Groningen, the Northern Provinces of the Netherlands; Fonds Economische Structuurversterking; Samenwerkingsverband Noord Nederland and Ruimtelijk Economisch Programma.

The generation and management of Genome-wide association study (GWAS) genotype data for the Lifelines Cohort Study are supported by the UMCG Genetics Lifelines Initiative and by a Spinoza Grant from the NWO, awarded to Cisca Wijmenga. The authors wish to acknowledge the services of the Lifelines Cohort Study, the contributing research centres delivering data to Lifelines, and all the study participants.

Raul Aguirre-Gamboa^1^, Patrick Deelen^1^, Lude Franke^1^, Jan A Kuivenhoven^2^, Esteban A. Lopera Maya^1^, Ilja M. Nolte^3^, Serena Sanna^1^, Harold Snieder^3^, Morris A. Swertz^1^, Judith M. Vonk^3^, Cisca Wijmenga^1^

^1^Department of Genetics, University of Groningen, University Medical Center Groningen, The Netherlands

^2^Department of Pediatrics, University of Groningen, University Medical Center Groningen, The Netherlands

^3^Department of Epidemiology, University of Groningen, University Medical Center Groningen, The Netherlands
