# Supplementary material for: Semi-automatic translation of medicine usage data (in Dutch, free-text) from Lifelines COVID-19 questionnaires to ATC codes
Source: Database (Oxford). 2023 Apr 26;2023:baad019. doi: 10.1093/database/baad019 (PMC10132814; doi:10.1093/database/baad019)
Supplement: baad019_Supp [file baad019_supp.zip › suppl_data/Supplementary Material 1 Table 1.docx]

| **ATC** | **GPK** | **GPK Omschrijving** | **HPK** |
| --- | --- | --- | --- |
| A01A | 57053 | Dentinox druppels go | 25593 |
| A01A | 123013 | Salicylzuur/rheumextract vlst oromucosaal | 14508 |
| A01A | 26387 | Mirre/lepelblad mondspoeling 40/80mg/ml | 249475 |
| A01AA01 | 2232 | Natriumfluoride tablet 0,56mg (0,25mg fluor) | 24686 |
| A01AA01 | 2232 | Natriumfluoride tablet 0,56mg (0,25mg fluor) | 425575 |
