# Supplementary material for: Semi-automatic translation of medicine usage data (in Dutch, free-text) from Lifelines COVID-19 questionnaires to ATC codes
Source: Database (Oxford). 2023 Apr 26;2023:baad019. doi: 10.1093/database/baad019 (PMC10132814; doi:10.1093/database/baad019)
Supplement: baad019_Supp [file baad019_supp.zip › suppl_data/Supplementary Material 1 Table 2.docx]

| **ATC** | **Original GPK Omschrijving** |  |
| --- | --- | --- |
| A01A | Dentinox druppels go | Dentinox |
| A01A | Salicylzuur/rheumextract vlst oromucosaal | Salicylzuur/rheumextract |
| A01A | Mirre/lepelblad mondspoeling 40/80mg/ml | Mirre/lepelblad |
| A01AA01 | Natriumfluoride | Natriumfluoride |
