# Supplementary material for: Semi-automatic translation of medicine usage data (in Dutch, free-text) from Lifelines COVID-19 questionnaires to ATC codes
Source: Database (Oxford). 2023 Apr 26;2023:baad019. doi: 10.1093/database/baad019 (PMC10132814; doi:10.1093/database/baad019)
Supplement: baad019_Supp [file baad019_supp.zip › suppl_data/Supplementary Material 2 Table 2.docx]

| Original Name | ATC | URL | Label | Name |
| --- | --- | --- | --- | --- |
| ANGELIQ TABLET FILMOMHULD | G03FA17 | http://purl.bioontology.org/ontology/UATC/G03FA17 | drospirenone and estrogen | ANGELIQ |
| ANGIOGRAF 650MG/ML FL 100ML | V08AA01 | http://purl.bioontology.org/ontology/UATC/V08AA01 | diatrizoic acid | ANGIOGRAF |
| ANGIOGRAFIN 650MG/ML FL50ML | V08AA01 | http://purl.bioontology.org/ontology/UATC/V08AA01 | diatrizoic acid | ANGIOGRAFIN |
| ANGIOX 250MG INJPDR FLACON | B01AE06 | http://purl.bioontology.org/ontology/UATC/B01AE06 | bivalirudin | ANGIOX |
