# Supplementary material for: Semi-automatic translation of medicine usage data (in Dutch, free-text) from Lifelines COVID-19 questionnaires to ATC codes
Source: Database (Oxford). 2023 Apr 26;2023:baad019. doi: 10.1093/database/baad019 (PMC10132814; doi:10.1093/database/baad019)
Supplement: baad019_Supp [file baad019_supp.zip › suppl_data/Supplementary Material 3 Table 1.docx]

| "Resource" | "Name" | "ATC" | "URI" | "Merknamen" |
| --- | --- | --- | --- | --- |
| "http://nl.dbpedia.org/resource/Cisplatine" | "Cisplatine" | "L01XA01" | "http://purl.bioontology.org/ontology/UATC/L01XA01" | "Cisplatine mayne , Platinol , Platistine , Platosin" |
| "http://nl.dbpedia.org/resource/Tenofovir_disoproxil" | "Tenofovir disoproxil" | "J05AF07" | "http://purl.bioontology.org/ontology/UATC/J05AF07" | "Reviro" |
| "http://nl.dbpedia.org/resource/Tenofovir_disoproxil" | "Tenofovir disoproxil" | "J05AF07" | "http://purl.bioontology.org/ontology/UATC/J05AF07" | "Viread" |
| "http://nl.dbpedia.org/resource/Foscarnet" | "Foscarnet" | "J05AD01" | "http://purl.bioontology.org/ontology/UATC/J05AD01" | "Foscavir" |
| "http://nl.dbpedia.org/resource/Idursulfase" | "Idursulfase" | "A16AB09" | "http://purl.bioontology.org/ontology/UATC/A16AB09" | "Elaprase" |
