# Supplementary material for: Semi-automatic translation of medicine usage data (in Dutch, free-text) from Lifelines COVID-19 questionnaires to ATC codes
Source: Database (Oxford). 2023 Apr 26;2023:baad019. doi: 10.1093/database/baad019 (PMC10132814; doi:10.1093/database/baad019)
Supplement: baad019_Supp [file baad019_supp.zip › suppl_data/Supplementary Material 5 Table 1.docx]

| Question |  |
| --- | --- |
| Question COVID24A1: “Have you taken any medications in the last 7 days?” | Multiple choice answers:  High blood pressure medicine (such as metoprolol, furosemide, enalapril), Inhaler, Corticosteroids in tablet form (such as prednisone), Other corticosteroids (such as injections, hormone creams, eye, or ear drops), Cholesterol lowering medication, Diabetes medication, Cough medicine, Pain medication, Other |
| Question COVID24A2:  "Which blood pressuring lowering medications (e.g., metoprolol, furosemide, enalapril) have you used in the last 7 days? Multiple answers are possible.” | Multiple choice answers: Hydrochlorothiazide, Furosemide (e.g., Lasix®), Bumetanide (e.g., Burien®), Atenolol, Metoprolol (e.g., Silken ZOC®), Bisoprolol (e.g., Emcor®), Captopril, Enalapril (e.g., Renitec®), Lisinopril (e.g., Zestril®), Nifedipine  Other: free text: |
| Question COVID24A3:  “Which inhalers have you used in the last 7 days? Multiple answers are possible.” | Multiple choice answers:  Salbutamol (e.g., Ventolin®, Airomir®), Formoterol (e.g., Oxis®, Foradil®), Salmeterol (e.g., Serevent®), Ipratropium (e.g., Ipraxa®, Atrovent®), Tiotropium (e.g., Spiriva®), Beclometasone (e.g., Qvar®), Budesonide (e.g., Pulmicort®), Fluticasone (e.g., Flixotide®), Foster®, Symbicort®, Seretide®  Other: free text: |
| Question COVID24A4:  “Which corticosteroids (such as prednisone) have you used in the last 7 days? Multiple answers are possible.” | Multiple choice answers:  Cortisone, Dexamethasone, Hydrocortisone, Prednisolone, Prednisone  Other: free text: |
| Question COVID24A5:  “Which other corticosteroids (such injections, hormone creams or eye/eardrops) have you used in the last 7 days? Multiple answers are possible.” | Multiple choice answers:  Injection with triamcinalonacetonide (e.g., Kenacort-A®), Salve or cream with triamcinolonacetonide, Neusspray met triamcinolonacetonide (e.g., Nasacort®), Eardrops with triamcinolonacetonide, Salve or cream with hydrocortisone, Salve or cream fluticasone (e.g., Cutivate®), Salve or cream with betamethasone, Salve or cream with dexamethasone, Eyedrops with dexamethasone, TriAnal®  Other: free text: |
| Question COVID24A6:  “Which cholesterol lowering medications have you used in the last 7 days? Multiple answers are possible.” | Multiple choice answers:  Simvastatin (e.g., Zocor®), Atorvastatin (e.g., Lipitor®), Fluvastatin (e.g., Lescol®), Rosuvastatin (e.g., Crestor®), Pravastatin, Gemfibrozil (e.g., Lopid®), Cholestyramine (e.g., Questran®), Ezetimib (e.g., Ezetrol®), Inegy®  Other: free text: |
| Question COVID24A7:  “Which diabetes-related medications have you used in the last 7 days? Multiple answers are possible.” | Multiple choice answers:  Insulin (e.g., Novorapid®, Novomix®, Insulatard®, Mixtard®, Lantus®), Metformin, Tolbutamide, Glibenclamide, Gliclazide (e.g., Diamicron®), Pioglitazone (e.g., Actos®), Repaglinide (e.g., NovoNorm®), Acarbose (e.g., Glucobay®), Sitagliptine (e.g., Yesnuvia®)  Other: free text: |
| Question COVID24A8:  “Which diabetes-related medications have you used in the last 7 days? Multiple answers are possible.” | Multiple choice answers:  Codeine, Noscapine, Broomhexine, Althea syrup of thyme syrup, Dextromethorphan, Pentoxyverine, Acetylcysteine, Carbocysteine, Promethazine, Chamomile or menthol  Other: free text: |
| Question COVID24A9:  “Which pain killers have you used in the last 7 days? Multiple answers are possible.” | Multiple choice answers:  Paracetamol (acetaminophen), Ibuprofen (e.g., Brufen®), Acetylsalicylic acid (e.g., Aspirin®), Diclofenac, Naproxen (e.g., Aleve®), Codeine, Tramadol (e.g., Tramal®), Oxycodone (e.g., OxyContin®, OxyNorm®), Morphine (e.g., MS Contin®, Oramorph®)  Other: free text: |
| Question COVID24A10:  “How many other different medicines have you used in the last 7 days? (maximum 5)” | free text: |
