# Supplementary material for: Semi-automatic translation of medicine usage data (in Dutch, free-text) from Lifelines COVID-19 questionnaires to ATC codes
Source: Database (Oxford). 2023 Apr 26;2023:baad019. doi: 10.1093/database/baad019 (PMC10132814; doi:10.1093/database/baad019)
Supplement: baad019_Supp [file baad019_supp.zip › suppl_data/Supplementary materials.docx]

# Supplementary materials

To build the ontology of drug names and their ATC codes, we downloaded data from several sources. What follows are uncurated examples of values from each of the sources, both as we received them and what we kept after some manual curation.

Attachment 1:

## Five examples of data from the Dutch Foundation for Pharmaceutical Statistics (Stichting Farmaceutische Kengetallen)

From the Dutch Foundation for Pharmaceutical Statistics, we downloaded a table that included the ATC code, the GPK, drug name and HPK number (https://www2.sfk.nl/producten/classificatie_index/atcboom/).

This data is relatively clean. Since the only information we needed was the name and ATC code of each drug, we discarded the GPK and HPK columns. We then removed the dosage information and combined duplicate entries. The application form of a drug sometimes plays a role for ATC codes, meaning it can make a difference whether something is a cream, tablet, injection or shampoo. It was often unclear whether to keep or remove this information as SORTA will take it as part of the drug name.

While cleaning the data from SFK, we reduced the number of unique entries from 30478 to 3713. This is how the curated version of the examples above looked after manual curation:

Attachment 2

## Examples from the database excerpt we received from IADB

To get access to the drug names and ATC codes from the G-Standaard, we requested a database excerpt with the relevant information from IADB. Here is what the uncurated data looked like:

It took some time to curate this data, and it may be that it is not always accurate. We also tried to match it with the data that we already had from the previous source(s), especially data from SFK. Therefore, we added additional columns. Below is a curated version of the example above.

Attachment 3

## Five examples from DBpedia

When creating an ontology, the reuse of predicates and URIs helps to keep different data sources compatible. For example, DBpedia offers many predicates to describe different kinds of relations. Therefore, it made sense to check which predicate DBpedia uses to refer to ATC codes. The Dutch version of the DBpedia uses two different predicates to describe the ATC code: <http://nl.dbpedia.org/property/atcSuffix> and <http://nl.dbpedia.org/property/atcPrefix>. Using that knowledge, we could query the ATC codes for all the drugs.

Just out of curiosity, to see how good the DBpedia results are compared to the data that we already collected, we cross-checked all the DBpedia entries with the data from SFK and IADB. The complete list of results can be found on GitHub at:

<https://github.com/AJKellmann/Scripts-for-the-translation-of-medicine-usage-data-to-ATC-codes/blob/main/Datasources%20to%20create%20Ontology/Dbpedia%20(Dutch)/Dutch%20Drugs%20and%20their%20ATC%20codes%20original.tsv>

The query we used to obtain those results is in the same GitHub repository:
<https://github.com/AJKellmann/Scripts-for-the-translation-of-medicine-usage-data-to-ATC-codes/blob/main/Datasources%20to%20create%20Ontology/Dbpedia%20(Dutch)/Original%20query%20to%20request%20drug%20names%20and%20ATC%20codes%20from%20Dbpedia.txt>

Attachment 4

## Our final data structure

Our first approach to represent the ATC codes as classes (owl:class) and the drugs as individuals did not work well. SORTA only takes the subclass relationship into account and ignores individual values. Therefore, we had to model the different ways to name the drug as a class–subclass relationship. This led us to start to building our ontology on top of the ATC code ontology from Bioportal. This ontology represents the ATC hierarchy as a tree structure. We added the drugs as leaves of the ATC code hierarchy’s tree structure. An excerpt of the resulting ontology is shown in **Figure 7**.

Attachment 5

## Relevant questions from the COVID-19 questionnaire:

The questions can also be found at https://wiki-lifelines.web.rug.nl/doku.php?id=medication_covid-19.
